# Health Literacy in Obstetric Patients: A Pharmacist’s Experience with The Newest Vital Sign

**DOI:** 10.3390/pharmacy3040372

**Published:** 2015-12-14

**Authors:** Denise Ragland, Nalin Payakachat

**Affiliations:** Department of Pharmacy Practice, University of Arkansas for Medical Sciences College of Pharmacy, 4301 W. Markham Street, #522, Little Rock, AR 72205, USA; E-Mail: npayakachat@uams.edu

**Keywords:** health literacy, pharmacist, Newest Vital Sign, obstetrics

## Abstract

Health literacy can greatly impact patients’ self-management of medical conditions and adherence to treatment recommendations. This study aimed to assess the health literacy of obstetric patients at a university-based women’s clinic using the Newest Vital Sign (NVS). A clinical pharmacist or student pharmacist utilized the instrument to interview women during a routine clinic visit. This project is a cross-sectional, retrospective study using the de-identified survey data. Descriptive statistics were used to provide a summary of the NVS scores in this population. The average age of the 140 participants was 27 years (SD = 6) with the range from 16 to 49 years old. The majority (78%) of the patients was ≤ 30 years old; 50% were white and 39% were black. The average NVS score was 3 (SD = 2.2). 49% had scores ≤ 3 which indicates limited literacy. This pilot study yielded preliminary data for future investigations of health literacy in this population while identifying a potential role for pharmacists. Due to its ease of use and quick administration, it is feasible to use the NVS instrument to routinely screen health literacy in an obstetric clinic setting.

## 1. Introduction

Clinical pharmacists contribute a myriad of services in the supervision of obstetric patients including in-patient drug therapy management of diabetes and hyperemesis gravidarum, pharmacokinetic-based treatment of acute infections, anticoagulant dosing, hyperalimentation, pharmacokinetic-based dosing of digoxin for fetal arrhythmias, and treatment of HIV-positive mothers and their offspring [1]. Ambulatory care services include co-management of chronic diseases (diabetes, anticoagulation, asthma, epilepsy, psychiatric conditions), pregnancy-related issues (anemia, constipation, nausea and vomiting), immunizations, screening and treatment of depression, contraception counseling, diabetes education, smoking cessation counseling, treatment of sexually transmitted infections, and providing relevant drug information in pregnancy and lactation. This report describes another service in which pharmacists have an opportunity for involvement: that of assessing patients’ health literacy.

Community pharmacists, whether retail or clinic-based, are in a prime position to catch misunderstandings about medication use which happens as often as 50% of the time [2]. Some of the misunderstanding may be due to low health literacy which, according to the Institute of Medicine (IOM), is a widespread problem. The IOM defines health literacy as the degree to which individuals have the capacity to obtain, process, and understand basic information and services needed to make appropriate decisions regarding their health. The 2004 IOM report, *Health Literacy: a Prescription to End Confusion* [3], found that nearly half of all American adults have difficulty understanding and using health information. This report also states that “efforts to improve quality, reduce costs, and reduce disparities cannot succeed without simultaneous improvements in health literacy.”

This study aimed to assess the health literacy of obstetric patients at a university-based women’s clinic using the Newest Vital Sign (NVS). Although the Test of Functional Health Literacy in Adults (TOFHLA) has been widely used for assessing health literacy, it takes 18 to 22 mins to administer. The short form (S-TOFHLA) can take 7–10 mins to administer. According to a previous study, the NVS can be administered in approximately 3 mins and its sensitivity is as great as the TOFHLA’s for identifying those with inadequate health literacy [4]. The NVS is an interview-administered health literacy instrument that measures prose and numeracy literacy as well as comprehension skills and abstract reasoning. It contains six questions asked by the administrator while the subject reviews and interprets a food label for the answers. The NVS score ranges from 0 to 6 which categorizes individuals into three groups: 0–1 suggests high likelihood of limited literacy; 2–3 indicates the possibility of limited literacy; and 4–6 almost always indicates adequate literacy. This health literacy instrument has been demonstrated to be easy to use in the clinic setting [5].

## 2. Experimental Section

At an U.S. teaching university medical center, prenatal care is provided by a collaborative healthcare team including a pharmacist, medical residents, dieticians, advance nurse practitioners, attending Ob/Gyn physicians, and Maternal-Fetal Medicine specialists. This high-risk obstetrical university-based women’s clinic, with a largely low socio-economic status population, was the site for this project. In an effort to improve patient education efforts, the pharmacist decided to assess health literacy as a need assessment/quality improvement project. After reviewing different health literacy instruments, the NVS assessment tool was chosen due to its ease of use in the clinic setting. Although the NVS is available in Spanish, Spanish-speaking subjects were excluded because the research staff did not speak Spanish. Approximately 20% of the patients at this clinic speak only Spanish.

The surveys were administered by the clinic pharmacist or a student pharmacist under the supervision of the pharmacist. The data was collected as a part of routine practice in the clinic between July and August, 2013. The survey contained the NVS instrument, age, and race. It did not include any patient’s personal health information. This project report utilized the data resulting from the pharmacist-initiated quality improvement project. All data were de-identified. Descriptive statistics were used to provide a summary of the NVS scores in this population. Associations among age and race with NVS levels were tested using Chi-square test or Fisher’s exact test where appropriate.

## 3. Results and Discussion

**Figure 1:**
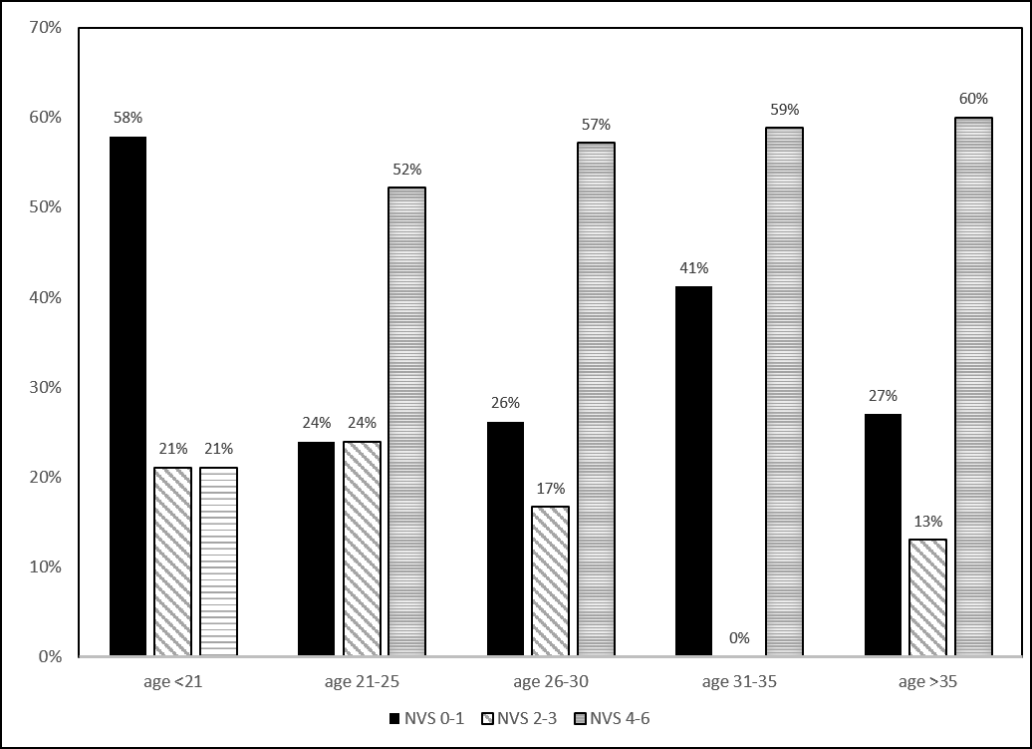
Newest Vital Sign scores by Age Groups.

A total of 140 patients completed the survey (Table 1). The average age was 27 years (SD = 6) with the range from 16 to 49 years old. The majority (78%) of the patients were young adults and teens 30 years old or younger. Half of the patients were white and another half were non-white (39% were black and 11% were other races). The average NVS score was 3 (SD = 2.2). 49% had scores ≤ 3 which indicates limited literacy. White patients had a higher proportion of adequate health literacy (NVS score of 0–1) when compared to non-whites (63% *vs.* 40%, *p* = 0.020). Young patients, age < 21 years old, had a higher proportion of limited health literacy (78.9%), compared to older groups (Figure 1, *p* = 0.046).

**Table 1:** Demographics and New Vital Sign scores of participants (*N* = 140).

	Total	NVS score			*p*-value
		0–1	2–3	4–6	
Age (years), mean ± SD (range)	27 ± 6 (16–49)				
< 21	19 (14%)	58%	21%	21%	0.046
21–25	46 (33%)	24%	24%	52%	
26–30	43 (31%)	26%	16%	58%	
31–35	17 (12%)	41%	0%	59%	
> 35	15 (11%)	27%	13%	60%	
Race					
White	70 (50%)	26%	11%	63%	0.022
Non-white	70 (50%)	37%	23%	40%	
NVS, mean ± SD (range)	3 ± 2.2 (0–6)				
0–1	44 (31%)				
2–3	24 (17%)				

NVS = Newest Vital Sign (score of 0–1 suggests high likelihood (50%) or more) of limited literacy; score of 2–3 indicates the possibility of limited literacy; score of 4–6 almost always indicate adequate literacy).

Our 49% limited health literacy rate is consistent with the IOM report that indicates nearly half of all American adults have difficulty understanding and using health information. Unfortunately, inadequate health literacy may result in poor health outcomes, especially in a high-risk obstetrical clinic. Not following treatment recommendations, often misconstrued as “noncompliance”, may be more related to the patients misunderstanding of medical information rather than indifference toward their health [6].

Limited health literacy is associated with less understanding of medical advice that can affect disease progression [7]. Additionally, adults with low literacy have more barriers to obtaining medical care, increased hospitalizations, and higher healthcare costs [8,9,10]. This issue not only affects an individual’s health but it also results in a significant economic burden. National estimates indicate that low health literacy costs the U.S. up to $238 billion each year [11]. Consequently, health literacy has become a national priority. Healthy People 2020 calls for a significant improvement in health literacy to advance the health of the population [12]. In addition, three federal initiatives also address this important topic. First of these is the *National Action Plan to Improve Health Literacy*, developed by the Department of Health and Human Services (HHS), along with over 700 public and private groups [13]. This plan outlines seven goals and strategies to improve the delivery of health information and healthcare services. Second, The *Plain Writing Act* requires all new federal government publications, forms, and publicly available documents to be written in a clear, concise, and well-organized manner [14]. Last but not least, the Affordable Care Act includes provisions that call for the need to clearly communicate health information, promote prevention, assure equality and cultural competencies, be patient-centered, and deliver high-quality care [15]. Pharmacists are in a unique position to address several of these issues and have a positive impact on patient outcomes.

Healthcare providers should be aware of patients’ health literacy so they can provide appropriate health education/instructions to better serve their population. Patients frequently have to make decisions about complicated medical conditions yet many highly-trained healthcare professionals may lack the skills or the time to communication clearly with patients. Pharmacists remain the most accessible health care professional for many [16]. As such they are in a prime position to ensure health care consumers have a clear comprehension of their disease states and understand how they can best optimize their health outcomes. In fact, lack of a personal connection with a pharmacist or pharmacy staff has been identified as a predictor of non-adherence [17].

In our experience, the NVS proved to be a quick and easy-to-use tool to assess health literacy. Although this project utilized the NVS in a clinic setting, it may also prove valuable in a retail pharmacy setting. Time required to administer the NVS in either setting could be offset by the time saved on call-backs from patients because of poor understanding of diagnosis or medications.

The results of this study will be used as a basis for discussion of health literacy with the medical staff that practice at this clinic site. Also, all written education materials used at this clinic are being reviewed by a plain language specialist. Last but not least, an interprofessional health literacy elective has been developed by the co-author. Health literacy education, training, and practice improvement tools can contribute to reorienting future and current pharmacists.

### Limitations

The generalizability of the results of this pilot study is limited by the small sample size and the fact that it was conducted at one clinic site only, which serves a generally low-income women referred from throughout the state of Arkansas.

## 4. Conclusions

This pilot project yielded preliminary data for future investigations of health literacy in this population while identifying a potential role for pharmacists. The fact that 49% had a NVS score which indicates limited health literacy, reinforces the need to review educational materials used in this clinic. Due to its ease of use and quick administration, we found it feasible to use the NVS instrument to routinely screen health literacy in an obstetric clinic setting.
